# MicroRNAs in lung cancer

**DOI:** 10.18632/oncotarget.20955

**Published:** 2017-09-16

**Authors:** Diana Castro, Márcia Moreira, Alexandra Monteiro Gouveia, Daniel Humberto Pozza, Ramon Andrade De Mello

**Affiliations:** ^1^ Department of Experimental Biology, Faculty of Medicine, University of Porto, Porto, Portugal; ^2^ Institute for Cellular and Molecular Biology (IBMC), Institute for Health Innovation, University of Porto, Porto, Portugal; ^3^ Faculty of Nutrition and Food Sciences, University of Porto, Porto, Portugal; ^4^ Department of Biomedical Sciences and Medicine, University of Algarve, Faro, Portugal; ^5^ Department of Medicine, Faculty of Medicine, University of Porto, Porto, Portugal

**Keywords:** microRNAs, lung cancer, inflammation, epithelial mesenchymal transition, interleukin 1

## Abstract

Lung cancer (LC) is a serious public health problem responsible for the majority of cancer deaths and comorbidities in developed countries. Tobacco smoking is considered the main risk factor for LC; however, only a few smokers will be affected by this cancer. Current screening methods are focused on identifying the early stages of this malignancy. Thus, new data concerning the roles of microRNA alterations in inflammation, epithelial-mesenchymal transition and lung disease have increased hope about LC pathogenesis, diagnosis, treatment and prognosis. MicroRNA mechanisms include angiogenesis promotion, cell cycle regulation by modulating cellular proliferation and apoptosis, and migration and invasion inhibition. In this context, this manuscript reviews the current information about many important microRNAs as they relate to the initiation and progression of LC.

## INTRODUCTION

Lung cancer (LC) is the most common cause of cancer death, with a high incidence and mortality in both genders. Despite progress in research regarding new targets, therapeutics, and strategies for LC screening and early diagnosis, prognosis is still poor, and overall survival rates remain low [1, 2]. LC can be divided in two types: small cell lung cancer (SCLC) with a neuroendocrine origin and non-small cell lung cancer (NSCLC). NSCLC accounts for approximately 80% of all LC and includes both squamous cell cancer and adenocarcinoma [2]. The main risk factor for LC is tobacco use; however, only a small percentage of smokers will develop LC (approximately 10% of all smokers), suggesting that other factors are also involved, such as individual genetic variations [3].

Recent data demonstrated the importance of regulatory mechanisms at the transcriptional level, such as gene regulation by small non-coding RNAs (microRNAs). These mechanisms include regulation of genes that mediate processes such as inflammation, the cell cycle, stress responses, differentiation, apoptosis and invasion. In this context, research regarding the involvement of microRNAs in LC tumorigenesis is increasing as the search for new biomarkers and therapeutic targets continues [1, 2]. Thus, the main objective of this brief review is to summarize the current information about the role of microRNAs in inflammation associated with the initiation and progression of LC.

### MicroRNAs

MicroRNAs are small, noncoding RNA molecules, 20-25 nucleotides in length, that negatively regulate gene expression at the post-transcriptional level. These molecules are encoded by specific genes and function in repressing mRNA translation or promoting mRNA degradation. MicroRNAs play an important role in many biological processes, such as inflammation, cell growth, apoptosis, development, differentiation, endocrine homeostasis and even cancer [4].

It is known that microRNAs are involved in lung inflammatory mechanisms, epithelial-mesenchymal transition, and, consequently, in LC development and therapy response. The potential applications of microRNAs in cancer diagnostics and prognostics, and as therapeutic targets have led to an increased interest in this research area [5]. The effects of microRNAs on cytokine signaling are based on transcription factors, cytokines and modulators of cytokine signaling. In addition, cytokine signaling is crucial in the differentiation of many immune cells. Thus, the role of microRNAs in immune cell differentiation is based on the regulation of cytokine expression and the regulation of their downstream signaling components. Several studies have shown that microRNAs, including miR-21, have an important role in balancing Th1 and Th2 responses to antigens [6, 7]. At present, the most studied microRNAs are miR-494, let-7, miR-155, miR-135b, miR-21, miR-125b, miR-196 and miR-210 [5].

miR-494 can be produced by lung cancer cells leading to tumor angiogenesis. In a hypoxic environment, this angiogenic process promotes tumor development through HIF-1α-induced upregulation of miR-494 [8]. On the other hand, miR-494 downregulates cellular proliferation in LC. It was demonstrated that constitutive expression of miR-494 in A549 lung cancer cells leads to the suppression of cell proliferation and induction of senescence. It was also demonstrated that insulin-like growth factor 2 mRNA-binding protein 1 (IGF2BP1) could be a target of miR-494. It was demonstrated that IGFBP1 has a role in carcinogenesis development and regulation by binding to mRNAs coding IGF2 (Insulin-like growth factor 2) and c-Myc [9]. In the A549 lung cancer cell line, miR-155 modulates cellular apoptosis and DNA damage through an Apaf-1-mediated pathway [10]. miR-153 inhibits the migration and invasion of human NSCLC by targeting ADAM19 and producing anti-tumor activity in LC through AKT suppression (Table 1) [11, 12].

**Table 1:** Key microRNAs in lung cancer

miRNAs	Gene targets	Biological mechanisms	References
**miR-494**	IGF2BP1	Promotes angiogenesis and decreases cellular proliferation	[8, 9]
**Let-7**	RAS, CDC25A, CDK6, cyclin D, LIN28, MYC, HMGA2, HOXA9, TGFBR1, BCL-XL, MAP4K3	Represses cell proliferation and regulates the cell cycle	[4, 13]
**miR-155**	hexokinase 2, APAf-1	Promotes glucose metabolism, modulates cellular apoptosis and DNA damage response	[10, 14]
**miR-153**	ADAM19, AKT	Inhibits the migration and invasion of human non-small cell lung cancer, inhibits proliferation and migration, and promotes the apoptosis of cultured lung cancer cells	[11, 12]
**miR-101**	COX-2, Lin28B, EZH2	Inhibits cell proliferation, inflammation, and dysregulation of the cell cycle	[15–18]
**miR-135b**	IL-1R1	Mediates the inflammatory response	[19]
**miR-200**	ZEB, E-cadherin, vimentin	Promotes EMT	[20, 21]
**miR-218**	Slug/ZEB2, tumor protein D52	Inhibits cell migration, invasion and EMT	[22, 23]
**miR-487b**	SUZ12, BMI1, WNT5A, MYC, KRAS	Represses the proliferation and invasion of LC cells	[24]

Another microRNA involved in cell proliferation and survival pathways, which is frequently altered in tumors, is Let-7. This microRNA is overexpressed during cell cycle progression and functions as a key regulator of several genes involved in cell proliferation. It is also known that reduced expression of the Let-7 family molecules in LC is associated with a poor survival rate. Furthermore, Let-7 directly regulates several proto-oncogenes involved in cell cycle regulation, such as RAS, CDC25A, CDK6 and cyclin D [13]. Thus, Let-7 controls cell proliferation by impairing the G1 to S transition [4].

### MicroRNAs and epithelial-mesenchymal transition (EMT)

EMT is a complex process that allows a polarized epithelial cell to go through several biochemical changes, ultimately assuming a mesenchymal cell phenotype. These changes increase cell migratory capacity, invasiveness, resistance to apoptosis and production of extracellular matrix components. Thus, EMT is crucial for epithelial cancer invasion and metastasis [25]. A strong correlation between EMT and the migratory and invasive capacity of tumor cells has been demonstrated. These mesenchymal-like cancer cells and TGF-β-induced EMT cells are characterized by increased invasive abilities compared to epithelial-like cancer cells. Thus, EMT is a key factor in the facilitation of tumor migration and invasion [26].

Several microRNAs have been described as important regulators of EMT, and their dynamic roles in the balance between EMT and the reverse process, termed mesenchymal to epithelial transition (MET), are recognized. One of these microRNAs is miR-153, which is downregulated in the TGF-β-induced mesenchymal phenotype of epithelial cancer cells [25].

Additionally, ectopic expression or targeted ‘knockdown’ of miR-153 resulted in downregulation or increased expression of SNAI1 (Snail Family Zinc Finger 1) and ZEB2 (Zinc Finger E-Box Binding Homeobox 2) protein levels, respectively. These two transcription factors promote the repression of the adhesion molecule E-cadherin to regulate EMT during embryonic development. In fact, SNAI1 and ZEB2 are considered critical pro-metastatic factors for their EMT-inducing capabilities [26, 27]. These studies also defined SNAI1 and ZEB2, both of which serve as transcriptional repressors of E-cadherin through binding with E-box elements in the E-cadherin promoter, as direct targets of miR-153. Therefore, E-cadherin is a key factor in EMT since its expression is decreased in the cells that undergo EMT in the presence of miR-153 inhibitors. Thus, the downregulation of miR-153 is crucial for the acquisition or maintenance of mesenchymal cell morphology and contributes to the EMT-associated carcinoma cell invasion induced by TGF-β [26].

Regarding the miRNAs that are related to the promotion of EMT and development of LC, the downregulated miR-200 family controls transcriptional factors such as Zinc Finger E-Box-Binding Homeobox (ZEB), E-cadherin and vimentin [20]. miR-218 also has an important role in the regulation of EMT-related traits and the metastasis of LC, in part by modulating Slug/ZEB2 signals [23] (Table 1). Increasing evidence has shown other miRNAs, including miR-124, miR-135a, miR-148a and miR-193a-3p/5p, to be powerful suppressors of EMT that are often downregulated in LC (for review see [28]).

### Cytokines and inflammatory cells, inflammation and lung cancer

**Figure 1:**
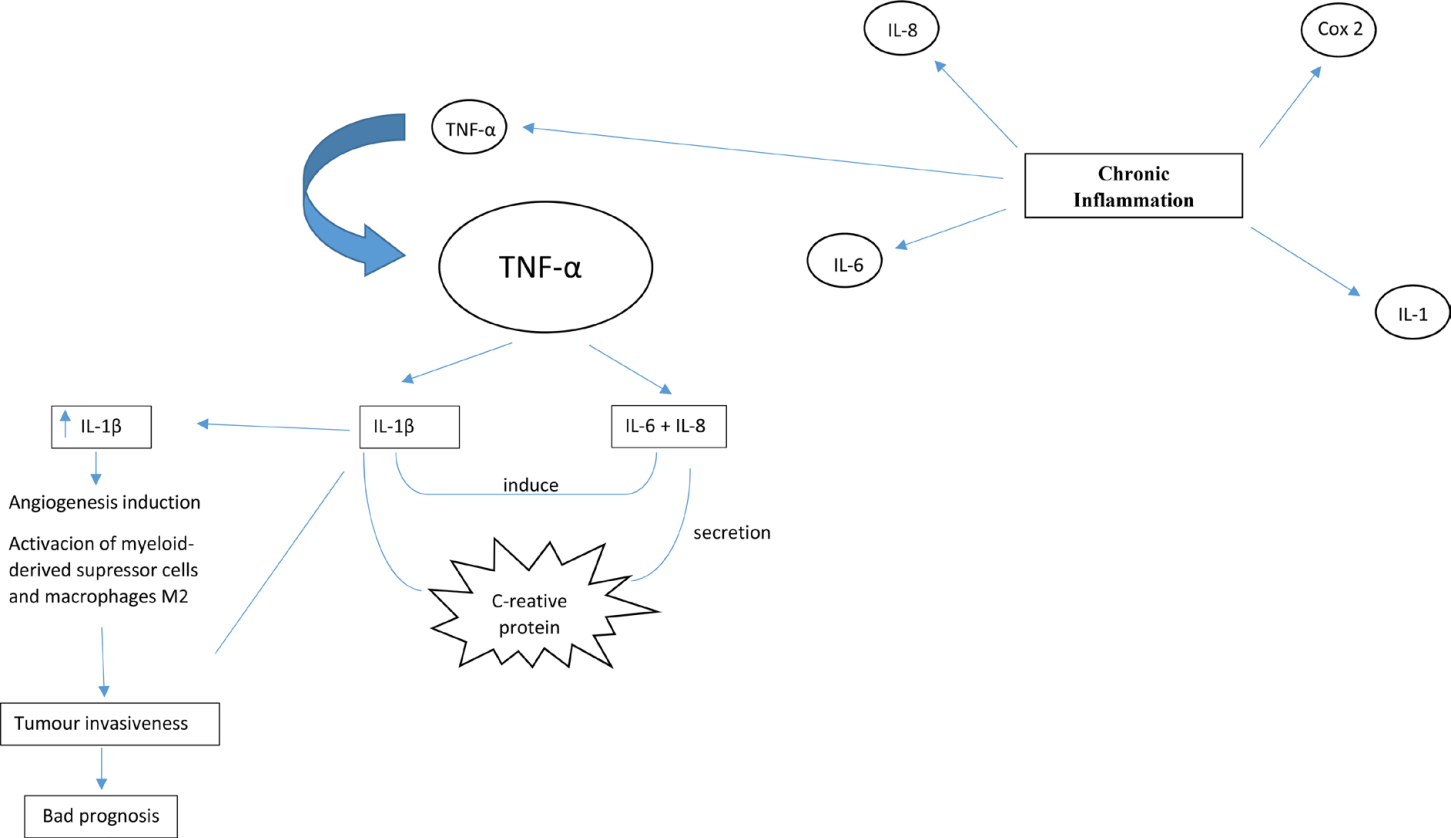
Chronic inflammation, a key promoting factor of lung tumorigenesis, is associated to secretion of cytokines including tumour necrosis factor α (TNF-α), interleukin 1 (IL-1), IL-6 and IL-8, and molecules such as cyclooxygenase-2 (COX-2) that are defined as “alarm cytokines” TNF-α is determinant to initiate and regulate the cytokine cascade by triggering the release of IL-1β and IL-6. IL-6 and IL-8 play different roles at a systemic level, being both inducible by IL-1β. IL-6 stimulates secretion of C-reactive protein that is an important inflammatory biomarker. High levels of IL-1β in the tumour microenvironment is directly associated with bad prognosis, mainly because IL-1β promotes tumour invasiveness by angiogenesis induction, activation of myeloid-derived suppressor cells and macrophages type M2.

Chronic inflammation, a key promoting factor of lung tumorigenesis, is associated with the secretion of cytokines, including tumor necrosis factor α (TNF-α), interleukin (IL) 1, IL-6, IL-8, and molecules such as cyclooxygenase-2 (COX-2), that are defined as “alarm cytokines” due to their roles in the initiation of inflammation. These cytokines are produced by normal cells, tumor cells and cellular components of the tumor microenvironment [5, 29]. TNF-α serves as an important factor in the initiation and regulation of the cytokine signaling cascade by triggering the release of IL-1β and IL-6 [29]. IL-1β is a pro-inflammatory cytokine that belongs to the interleukin-1 family, which is composed of several members, including IL-1α and IL-1R antagonist (IL-1Ra), an inhibitor of preformed IL-1β. It was demonstrated that a variable number of the IL-1Ra gene is not an independent risk factor for NSCLC, but it can play a role in prognosis when combined with polymorphisms of the IL-1β gene [3]. IL-6 and IL-8 play different roles at the systemic level, and both are inducible by IL-1β [30]. IL-6 stimulates secretion of C-reactive protein, an important inflammatory biomarker (Figure 1).

IL-1β is also directly involved in the regulation of plasma levels of C-reactive protein by gene regulation and indirectly involved through the production of several pro-inflammatory molecules, including COX-2, inducible nitric oxide synthase, and IL-6, among other cytokines. High levels of IL-1β in the tumor microenvironment are directly associated with a poor prognosis mainly because IL-1β promotes tumor invasiveness by angiogenesis induction and the activation of myeloid-derived suppressor cells and M2 macrophages [29, 31].

IL-1β inhibits miR-101, a tumor-suppressive microRNA, via the COX-2-HIF1α pathway [17]. The role of COX-2 in the initiation and progression of NSCLC is already recognized. It was demonstrated that knockdown of COX-2 significantly increased miR-101 expression, showing that COX-2 negatively controls miR-101 expression in NSCLC cells. Previous studies also showed that IL-1β activates HIF1α through the NF-κB/COX-2 pathway. HIF1α is a transcriptional repressor for miR-101 via IL-1β interactions in NSCLC. It was demonstrated that IL-1β promotes the activation of NF-κB, which transcriptionally activates *Lin28B.* This protein coding gene represents a key link the inflammation associated with cancer cell transformation and is a novel target of miR-101. Thus, *Lin28B* is upregulated by repression of miR-101 (IL-1β). It was concluded that downregulation of miR-101 by IL-1β is a key mechanism in the promotion of carcinogenesis and the development of malignant processes [5, 18].

miR-101 is also related to IL-1β via Enhancer of Zeste 2 Polycomb Repressive Complex 2 Subunit (EZH2), a member of the polycomb-group family that form multimeric protein complexes, including the complex involved in the methylation of histone H3. Several studies demonstrated the upregulation of EZH2 in LC, and it is postulated that this upregulation promotes tumor development and progression by dysregulation of the cell cycle [15]. The first study that proposed the role of the IL-1β/miR-101/EZH2 axis in LC found that autocrine and paracrine IL-1β stimulated the downregulation of miR-101 in a Xuanwei LC cell line (XWLC-05), leading to EZH2 upregulation, which in turn triggered tumorigenesis [16].

miR-135b expression is regulated by IL-1R1, a direct target of miR-135b, during IL-1R1/IL-1α-mediated inflammation. By using IL-1R1 knockout mice, it was demonstrated that miR-135b expression is IL-1R1 dependent. Furthermore, *in vitro* activation of the IL-1R1 pathway in mouse embryonic fibroblasts and lung epithelial cells resulted in increased miR-135b levels. Thus, there is a negative feedback loop in which IL-1R1 and miR-135b self-regulate one another. In addition, to decrease the inflammation induced by cigarette smoke, miR-135b regulates IL-1R1 expression by targeting its downstream mediators, Caspase-1 and IL-1β [19].

### Cigarette smoke: alterations on microRNAs

The identification of differentially expressed microRNAs between the non-malignant tissues of current smokers and the non-malignant tissues of individuals who had never smoked suggests that smoking history plays an important role in microRNA expression. It was hypothesized that the altered expression of microRNAs in the non-malignant tissues of current smokers affects distinct cellular pathways and may be an early event in smoking-associated tumorigenesis [32]. Finding the same pattern of differentially expressed microRNAs may differentially influence LC prognosis and may represent an important marker for therapeutic intervention. For example, in a specific smoking status group, miR-195, miR-138 and miR-150 demonstrated aberrant and recurrent expression, and were significantly associated with the survival rate of this group [32].

Experimental data provided evidence that exposure to various environmental or lifestyle factors, such as environmental cigarette smoke, result in extensive alterations to miRNA expression in the lung. The expression of 484 miRNAs was analyzed in rat lungs after exposure to environmental cigarette smoke for 28 days, which led to the downregulation of 126 of these miRNAs [33]. Most miRNAs are downregulated in tumors when compared to normal tissues because of the association between microRNA levels and cellular differentiation. Thus, the reduction of microRNA expression in cancer cells is associated with their degree of cellular differentiation, and consequently, the reduction is greater in differentiated tumors [34]. The most notably downregulated microRNAs belong to the families of let-7, miR-10, miR-26, miR-30, miR-34, miR-99, miR-122, miR-123, miR-124, miR-125, miR-140, miR-146, miR-191, mi-192, miR-219, miR-222 and miR-223. These microRNAs are responsible for a variety of cell functions, including apoptosis, proliferation, angiogenesis, gene expression and stress response.

It was demonstrated that increasing or decreasing the expression of miR-218, one of the microRNAs in human airway epithelium most commonly affected by smoking, was sufficient to induce a respective change in the expression of predicted miR-218 mRNA targets in both primary bronchial epithelial cells and H1299 cells. On the other hand, the alteration of miR-218 expression may influence the expression of MAFG gene targets since binding sites for MAFG are overrepresented in the epithelial cells of smokers [34]. miR-294, an inhibitor of transcriptional repressors, is also affected; it is upregulated in an environment with cigarette smoke [33].

When human airway epithelial cells are exposed to cigarette smoke condensate, epigenetic repression of miR-487b expression occurs. This downregulation, together with increased expression of miR-487b oncogenic targets (SUZ12, BMI1, WNT5A, MYC and KRAS), leads to increased proliferation and invasion in LC cells. Thus, the repression of miR-487b increases tumorigenesis, proliferation and invasion, and the expression of this microRNA inhibits the growth and metastatic potential of LC [24]. Furthermore, tobacco smoke carcinogens can also generate epigenetic silencing, for example, the downregulation of miR-200 and miR-205 through epigenetic mechanisms, to induce EMT; in these cases, EMT was strongly associated with LC [21].

Recently, it has been thought that the downregulation of microRNAs induced by cigarette smoke could be reversed by the oral administration of chemopreventive agents (*e.g*. N-acetylcysteine, oltipraz, indole-3-carbinol, 5,6-benzoflavone and phenethyl isothiocyanate) [33]. According to the authors, these agents could modulate proliferation, apoptosis, differentiation, angiogenesis or p53 functions. Furthermore, some human polymorphic microRNAs that are downregulated by cigarette smoke can be protected by these chemopreventive agents. For example, phenethyl isothiocyanate can affect the downregulation of some of the miRNAs that participate in a variety of functions, including the stress response (miR-125b), NF-kB activation (miR-146-prec), TGF-β expression (miR-26a), Ras activation (let-7a, let-7c and miR-192), cell apoptosis (miR-99b), cell proliferation (let-7a, let-7c and miR-222-prec) and angiogenesis (let-7a, let-7c, miR-123-prec and miR-222-prec). The efficacy of these agents may be influenced by genetic polymorphisms in these miRNAs. The optimal chemopreventive agents should not modify the baseline expression of genes and should be able to counteract the molecular alterations induced by carcinogens, re-establishing a normal physiologic situation [35].

### MicroRNAs: a brief reference to diagnosis and prognosis

Early diagnosis and the adequate treatment of each patient with LC are essential in order to improve clinical outcomes. Consequently, there is an urgent need to identify minimally invasive biomarkers to facilitate early diagnosis. Interestingly, microRNAs can be found in the nucleus of the cells and in blood. The discovery of microRNAs, namely, circulating miRNAs, sheds new light on tumor diagnosis and prognosis [36, 37].

Plasma samples from 100 early stage NSCLC patients and 100 non-cancer controls were screened for 754 circulating microRNAs via qRT-PCR using TaqMan microRNA arrays. The results revealed that a group of 24 miRNAs were significantly and independently associated with LC development and with predicting and establishing risk factors [38]. However, it was shown that a group of six microRNAs (miR-30c, miR-616, miR-146b-3p, mi-566, miR-550 and miR-939) were substantially increased, and another two miRNAs (miR-339-5p and miR-656) were substantially diminished in the serum of LC patients. The increased miRNAs are particularly relevant in the earlier stages of disease, suggesting their importance for early diagnosis [37].

Plasma miR-195 could be used as a biomarker for the early detection and as an independent unfavorable prognostic factor for NSCLC since it is downregulated in patients with this pathology when compared with healthy controls [39]. It was also observed that 10 miRNAs had a significantly different expression level in serum of cases with NSCLC when 400 NSCLC cases and 220 controls were analyzed. Thus, the combination of multiple serum miRNAs allows a more accurate cancer diagnosis [36].

It was reported that increased plasma levels of miR-let-7b can be an indicator of survival. Therefore, decreases in the plasma expression of let-7b were associated with worse prognosis and poorer survival. The reduction in serum miR-223 expression was also associated with poor survival outcomes in TNM stage I patients. These findings showed that LC patients with epigenetic alterations were predisposed to more aggressive disease [40]. Considering that Let-7 can clinically increase the postoperative survival of patients with LC by suppressing tumor proliferation and survival through the mediation of oncogenes and other cell functions, it has been one of the main potential therapeutic targets studied in cancer therapy. Inflammation is one of the mechanism clinically affected by Let-7 expression in cancer. Molecules related to inflammation, such as NFκB, have are involved in the regulatory feedback loop controlling Let-7 expression in inflammation and cancer. Positive feedback occurs when NFκB reduces let-7 levels, inhibiting IL-6 expression and consequently activating NFκB [13].

Additionally, microRNAs can also be used to predict the risk of radiation-induced esophageal toxicity in patients receiving radiochemotherapy for NSCLC. High serum miR-155 and miR-221 levels during the first two weeks of radiochemotherapy were associated with the development of severe radiation esophagitis. Thus, these miRNAs may be useful as important predictors for this form of toxicity [41].

## CONCLUSIONS

Despite the high rates of morbidity and mortality related to lung cancer (LC), there is still no good early stage LC screening. The new data about the roles of microRNA alterations in lung disease, including LC, bring new hopes for the pathogenesis, diagnosis, treatment and prognosis of this cancer. Although tobacco smoking is the main risk factor, there are some cases of LC in non-smokers, and only a small percentage of smokers will develop LC. Thus, there is likely an association of both environmental and genetic factors. MicroRNA alterations may play a crucial role in lung inflammation and epithelial-mesenchymal transition. The levels of serum microRNAs could be employed as cancer markers and used in the diagnosis of early stages of the disease; they could also be used to predict prognosis, maximizing the efficiency of treatment. Chemopreventive agents that act in these microRNA alterations can serve as new therapeutic targets in some patients with LC in the future. Finally, more studies are needed to demonstrate the influence of microRNAs in lung inflammation and their use in disease screening to improve the quality of life and to decrease the mortality of LC patients.
